# Effect of Variation in Extended Depth-Of-Focus Design on Myopia Control and Visual Function in Japanese Children

**DOI:** 10.1097/ICL.0000000000001294

**Published:** 2026-06-25

**Authors:** Sayuri Ninomiya, Shizuka Koh, Sachiyo Ueshima, Kohji Nishida

**Affiliations:** Itami Central Eye Clinic (S.N., S.U.), Hyogo, Japan; Department of Innovative Visual Science (S.K.), The University of Osaka Graduate School of Medicine, Osaka, Japan; and Department of Ophthalmology (S.K., K.N.), The University of Osaka Graduate School of Medicine, Osaka, Japan.

**Keywords:** Myopia control, Extended depth-of-focus, Soft contact lenses, Japanese children, Visual function, Optical design

## Abstract

**Objectives:**

To evaluate the efficacy of extended depth-of-focus (EDOF) soft contact lenses with different optical profiles in controlling myopia progression and their effect on visual function in Japanese children.

**Methods:**

In this 12-month multicenter, double-masked, randomized controlled trial, 60 Japanese children aged 6 to 12 years were assigned to wear two types of EDOF soft contact lenses with different depths of focus (Mid-EDOF [+1.50 D] or Low-EDOF [+0.75 D]) or single-vision daily disposable contact lenses (control). Axial length and cycloplegic spherical equivalent (SE) were measured at 6 and 12 months. Visual function, including contrast sensitivity, higher-order aberrations (HOAs), and subjective visual quality, was assessed at baseline and follow-up.

**Results:**

Mid-EDOF lenses significantly suppressed axial elongation and SE progression by 49% and 59%, respectively, compared with single-vision lenses (*P*<0.01). Low-EDOF lenses exerted no significant effect on axial elongation or SE progression. Visual function assessments revealed reduced contrast sensitivity and increased HOAs in the Mid-EDOF group; subjective visual quality remained comparable across groups. No serious adverse events were reported.

**Conclusion:**

Mid-EDOF lenses significantly suppress myopia progression, with tolerable visual function changes, potentially providing effective myopia control. Low-EDOF lenses showed no significant benefit over single-vision lenses. EDOF lenses' optical profile critically affects treatment efficacy.

Myopia is a growing global health concern, whose prevalence has surged dramatically in recent decades, particularly in East and Southeast Asia. In countries such as China and South Korea, the prevalence of myopia in elementary school children exceeds 80%.^1^ In Japan, the prevalence of myopia is also high, particularly in urban areas.^2^ Yotsukura et al.^3^ reported that 76.5% of elementary school students and 94.9% of junior high school students in Tokyo, Japan, met the criterion for myopia (spherical equivalent [SE] ≤ −0.50 D), of whom 4.0% and 11.3%, respectively, had high myopia (≤−6.00 D). High myopia poses a significant risk for vision-threatening conditions such as glaucoma, retinal detachment, and myopic maculopathy.^4–6^ As myopia is projected to affect nearly half of the world's population by 2050^7^ and the need for treatment and services of myopia-related complications increases, the financial burden on health care systems could rise substantially.

Myopia progression is influenced by a combination of genetic and environmental factors, including time spent outdoors, near work, and age at onset.^8–10^ Clinically, early-onset myopia in children is particularly alarming because of its strong association with rapid progression and an elevated risk of high myopia.^11–13^ Numerous interventions have been proposed to retard myopia progression in children, including low-dose atropine eye drops,^14–16^ orthokeratology,^17–19^ spectacle lenses incorporating specialized optical designs,^20,21^ and multifocal soft contact lenses^22^ designed with progressive addition optics,^23^ concentric or dual-focus zones,^24,25^ or extended depth-of-focus (EDOF) profiles.^26,27^

One proposed mechanism of optical myopia control involves the use of relative peripheral plus power to counteract peripheral hyperopic defocus, which is believed to stimulate axial elongation.^28–30^ In prolate-shaped myopic eyes, single-vision correction typically focuses on-axis light on the macula, whereas peripheral light may be focused behind the retina. Optical designs that introduce greater plus power in the periphery, such as multifocal and EDOF lenses, may help shift these peripheral focal points forward, thereby reducing the stimulus for ocular growth.^31,32^ Clinical studies have demonstrated variable efficacy across optical strategies. Effect sizes range from negligible to 30% to 50% for spectacle lenses, 30% to 60% for orthokeratology, and 21% to 55% for multifocal soft contact lenses.^33,34^ Such variability may be partly attributed to the heterogeneity in study populations, including age and ethnicity.

Extended depth-of-focus lenses aim to reduce peripheral hyperopic defocus and introduce a controlled level of retinal blur, both of which are thought to suppress axial elongation. In recent years, several studies conducted in Asian countries including China, Taiwan, India, and Japan have suggested that EDOF lenses can retard myopia progression and axial elongation.^26,27,35,36^ However, the reported effects vary, and the optimal design parameters for maximizing efficacy remain uncertain. Extended depth-of-focus profiles with a greater depth of focus may provide stronger myopia control but raise concerns about compromised visual quality. In contrast, lower depth of focus profiles may preserve visual function but offer weaker efficacy. Thus, the balance between the treatment efficacy and visual performance is still under discussion. Optical myopia-control strategies differ not only in efficacy but also in their effect on visual performance. In particular, designs that increase retinal defocus or extend depth of focus may improve myopia control at the cost of reduced contrast sensitivity or subjective visual clarity. Therefore, comparative evaluation of both efficacy and visual function is clinically important when interpreting the translational value of EDOF lens designs. However, detailed characterization of the nature of differences in power distribution of specific EDOF optical profiles and their functional implications is limited.

To overcome these uncertainties, the present study was designed with two objectives: (1) to evaluate the efficacy of EDOF soft contact lenses in slowing myopia progression in Japanese children and (2) to examine whether different EDOF designs (low vs. mid) produce differential treatment outcomes in axial elongation, refractive progression, and visual function.

## METHODS

This prospective, multicenter, randomized, double-masked, parallel-group clinical study was reviewed and approved by the institutional review board and ethics committee of The University of Osaka Hospital (registration number: S20004). The study adhered to the principles of the Declaration of Helsinki. Written informed consent was obtained from the parents or legal guardians of all participants. Consent was also obtained from the children themselves using age-appropriate explanation procedures.

### Participants

Children aged 6 to 12 years with a cycloplegic SE refractive error between −1.00 D and −6.00 D and astigmatism ≤0.75 D were enrolled. All participants had a corrected distance visual acuity of 20/25 or better in both eyes. The exclusion criteria included manifest strabismus, any ocular or systemic condition affecting contact lens wear, and history of myopia-control interventions and use of medications known to influence accommodation. Although measurements were performed for both eyes, only data from the left eye were used for statistical analysis to avoid intrasubject correlation.

### Types of Contact Lenses

**FIG. 1.**
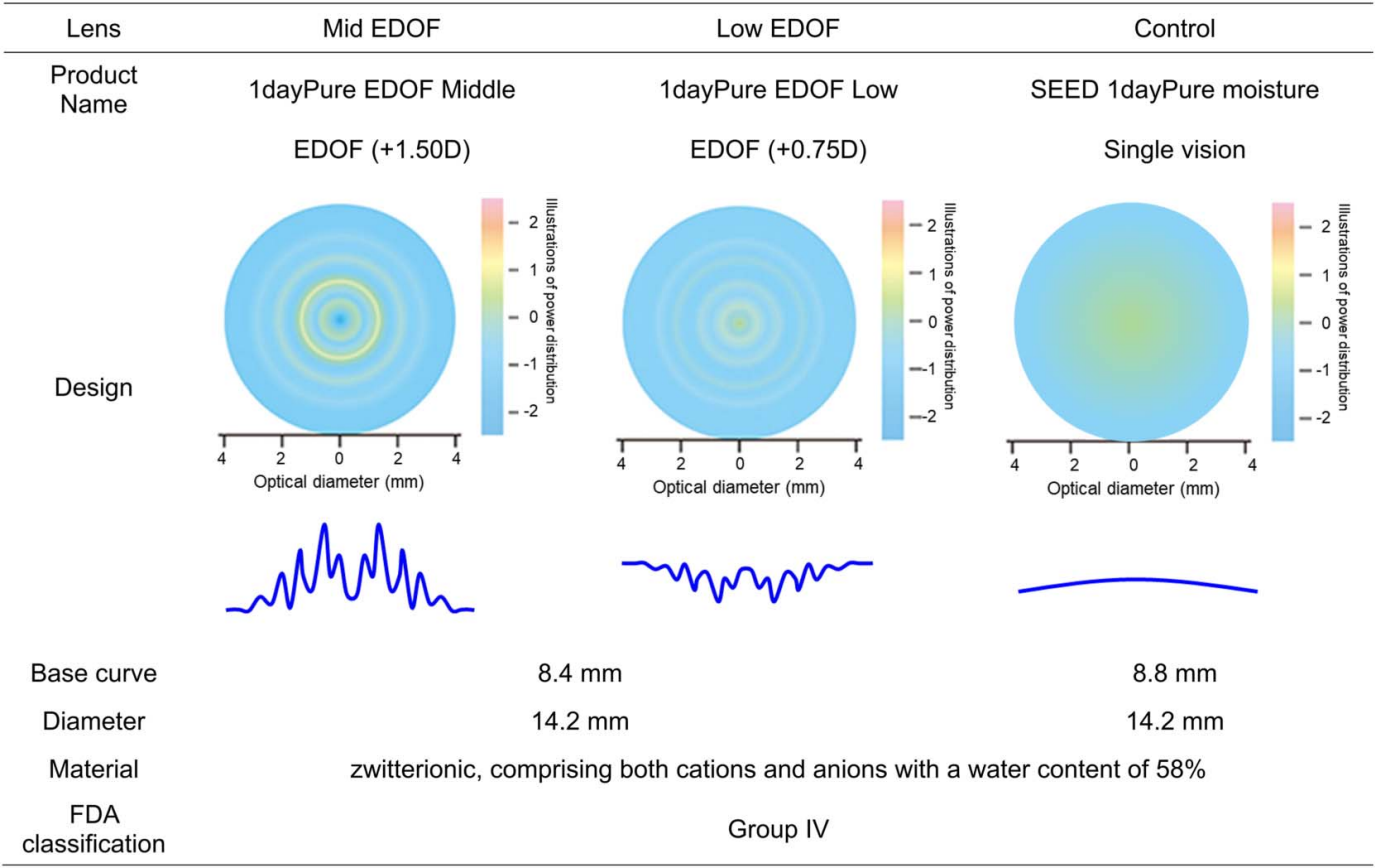
The optical designs of the tested lenses. EDOF, Extended depth-of-focus; FDA, Food and Drug Administration.

Two types of daily disposable EDOF soft contact lenses with different depths of focus—Mid-EDOF and Low-EDOF—were tested in this study (1 dayPure EDOF Middle and 1 dayPure EDOF Low; SEED Co., Ltd, Tokyo, Japan; hereinafter referred to as Mid-EDOF and Low-EDOF, respectively). The two EDOF lenses were identical in material and design specifications, except for the depth of focus, which was +1.50 D for Mid-EDOF and +0.75 D for Low-EDOF lenses. The optical designs of the lenses and their power distribution profiles are presented in Figure 1. Both EDOF lenses were based on an annular, nonmonotonic power distribution intended to extend the depth of focus, with the Mid-EDOF design exhibiting a greater magnitude of power variation across the optical zone compared with the Low-EDOF design. A daily disposable single-vision soft contact lens (SEED 1 dayPure moisture; SEED Co., Ltd) was used as the control. The material of this lens was identical to that of the EDOF lenses but had a single-vision design (hereinafter referred to as the control).

### Experimental Protocol

Eligible participants provided informed consent at the screening visit and underwent baseline examinations. Using a web-based system, they were randomly assigned to the Mid-EDOF, Low-EDOF, or control group in a 1:1:1 ratio, with allocation stratified by age, baseline refraction, and clinical site. Following lens fitting and handling instructions, lens wear was initiated for a maximum of 6 hr on the first day and subsequently increased to at least 8 hr per day for a minimum of 5 days per week throughout the 12-month study period.

Lens fitting performance was assessed at follow-up visits using slit lamp biomicroscopy, including evaluation of lens centration and overall fitting characteristics. Measurements including the cycloplegic SE, axial length (AL), and subjective evaluations (comfort, vision, and compliance) were performed at baseline, 6, and 12 months. Participants underwent visual function testing (contrast sensitivity, higher-order aberrations [HOAs], and subjective visual quality) once within 3 months of starting lens wear. Safety monitoring was performed by measuring the corneal endothelial cell count at baseline and 12 months.

### Measurement Methods

Cycloplegic SE was measured using an autorefractometer (TONOREF II, NIDEK, Aichi, Japan). The AL was measured using noncontact optical biometry (OA-2000, Tomey Corporation, Aichi, Japan). Subjective evaluations of comfort, vision, and compliance were achieved using structured questionnaires. Contrast sensitivity was evaluated under photopic conditions using the CSV-1000E chart (VectorVision, Greenville, OH) at a viewing distance of 2.5 m with best spectacle correction, and the area under the log contrast sensitivity function (AULCSF) was calculated. Higher-order aberrations were measured using a Hartmann–Shack aberrometer (KR-1W; Topcon Corporation, Tokyo, Japan) through the natural pupil without dilation in a dark room. The wavefront data obtained for the central 4- and 6-mm pupils were expanded up to the sixth order of Zernike polynomials. Subjective visual quality was rated on a five-point scale under both photopic and mesopic lighting conditions.

### Study Endpoints

The primary endpoint of the study was the change in AL at 12 months. The secondary endpoints included AL change at 6 months, changes in cycloplegic SE at 6 and 12 months, and changes in visual function measures (contrast sensitivity, HOAs, and subjective visual quality).

### Statistical Analysis

The mean difference was estimated to be 0.09 mm with a SD of 0.08 mm. The significance level was adjusted using the Bonferroni method to account for two pairwise comparisons (single-vision vs. Low-EDOF lenses and single-vision vs. Mid-EDOF lenses). Accordingly, α was set at 0.025 to yield an adjusted *P*-value of approximately 0.05 [1 − (1 − α)^2^=0.05]. Assuming a power of 0.9 (1 − β=0.9), the required sample size for a two-sample comparison of means (unpaired) was calculated to be 21 participants.

For the comparison between Low-EDOF and Mid-EDOF lenses, previous studies from the Brien Holden Vision Institute have shown that the efficacy of myopia control does not differ between EDOF contact lenses with depths of focus of 1.50 and 1.25 D. These findings suggest that once the depth of focus exceeds a certain threshold, the degree of myopia control may plateau. Presuming no difference between depths of focus of 1.50 and 1.25 D, the expected mean difference in AL between Low-EDOF and Mid-EDOF lenses was assumed to be 0.06 mm, with a SD of 0.08 mm. Under this assumption, α=0.05, and a power of 0.8 (1 − β=0.8), the required sample size for an unpaired two-sample comparison was calculated to be 29 participants.

Changes in the AL and SE values under cycloplegia at visit one relative to baseline were compared among the groups using a mixed-effects model. The change from baseline was used as the dependent variable; group, visit, and the group-by-visit interaction were designated as fixed effects; and participant was set as a random effect. Based on this model, Bonferroni-adjusted least-squares means were estimated for each group, and the adjusted mean differences (with two-sided adjusted confidence intervals) for Low-EDOF and Mid-EDOF lenses relative to the control device group were obtained. Multiple regression analyses were also performed for the change in AL and objective refraction at visits six and eight relative to baseline (visit 1). The allocation group, baseline value, age, and sex (male/female) were included as explanatory variables.

## RESULTS

This study enrolled 60 participants, of whom 59 completed the 12-month follow-up. One participant withdrew before the 6-month visit because of scheduling conflicts (Table 1). During the study period, slitlamp examination revealed no apparent differences in lens fitting and lens centration among the groups. The mean daily wearing time across all groups was 12.9 hr, indicating generally good adherence to the prescribed wear schedule, although the shortest mean wearing time was 8.7 hr/day.

**TABLE 1.** Baseline Characteristics and Changes in the Axial Length and Spherical Equivalent

Visit	Mid			Low			Control			*P*
	Baseline	6 mo	12 mo	Baseline	6 mo	12 mo	Baseline	6 mo	12 mo	
n	21	20	20	22	22	22	17	17	17	
Age (yrs)	10.2±1.4			10.0±1.4			10.1±1.5			*0.823*
M:F	8:13			7:15			8:9			*0.624*
AL (mm)	24.65±0.89	24.80±0.87	24.90±0.89	24.73±0.69	24.91±0.73	25.07±0.75	24.63±0.73	24.83±0.71	24.99±0.72	*0.948*
SER (D)	−3.20±1.12	−3.46±1.10	−3.62±1.14	−3.28±1.23	−3.74±1.38	−4.07±1.37	−2.74±1.07	−3.15±0.88	−3.56±1.05	*0.277*
Change in AL (mm)		0.09±0.10	0.19±0.14		0.18±0.10	0.33±0.16		0.20±0.08	0.36±0.14	
Change in SER (D)		−0.18±0.33	−0.34±0.43		−0.46±0.29	−0.79±0.38		−0.40±0.36	−0.82±0.43	

AL, axial length; F, female; M, male; SER, spherical equivalent refractive error.

**FIG. 2.**
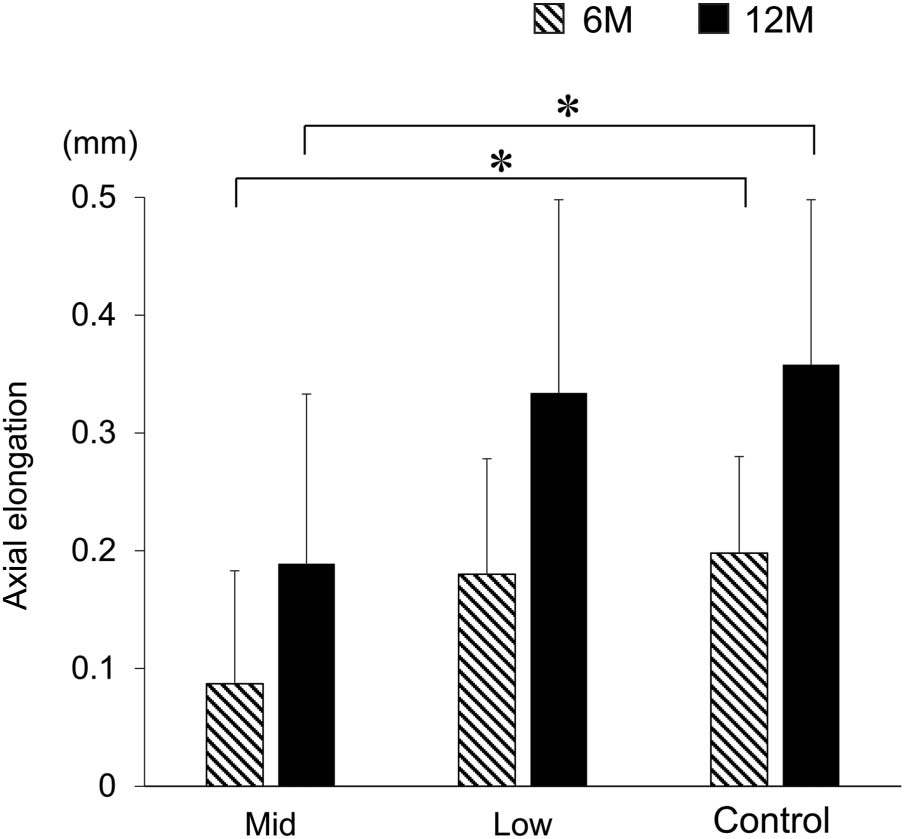
Changes in axial length. Axial elongation was significantly reduced in the Mid-EDOF group compared with the control group (0.17 mm; 49% reduction), whereas no significant difference was observed between the Low-EDOF and control groups. Mid: Mid-EDOF group, Low: Low-EDOF group. EDOF, Extended depth-of-focus.

At 6 months, the mean axial elongation was 0.09±0.10 mm in the Mid-EDOF group, 0.18±0.10 mm in the Low-EDOF group, and 0.20±0.08 mm in the control group. At 12 months, the corresponding values were 0.19±0.14 mm (Mid-EDOF), 0.33±0.16 mm (Low-EDOF), and 0.36±0.14 mm (control), respectively. Axial elongation in the Mid-EDOF group (0.17 mm) was significantly lower by 49% relative to the control group. No significant difference was observed between the Low-EDOF and control groups (*P*=0.19; Fig. 2).

**FIG. 3.**
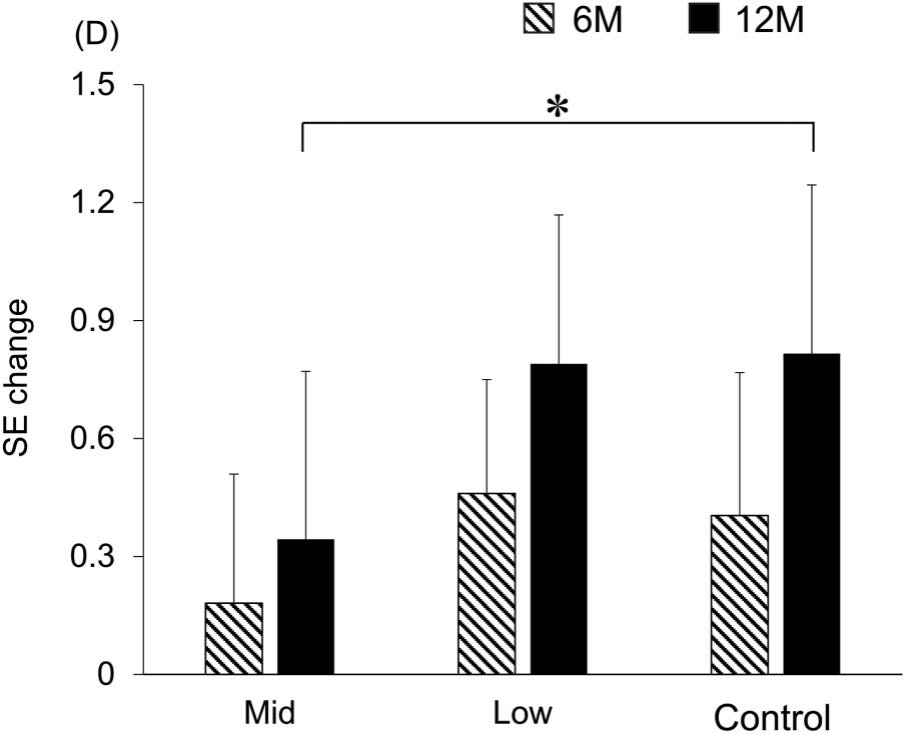
Changes in spherical equivalent refraction. The mean cycloplegic spherical equivalent changes at 6 and 12 months were significantly reduced in the Mid-EDOF group compared with the control group, corresponding to a 59% (0.48 D/year) suppression, whereas no significant difference was observed between the Low-EDOF and control groups. Mid: Mid-EDOF group, Low: Low-EDOF group. EDOF, Extended depth-of-focus.

The mean change in cycloplegic SE between baseline and 6 months was −0.18±0.33 D (Mid-EDOF), −0.46±0.29 D (Low-EDOF), and −0.40±0.36 D (control). At 12 months, progression was−0.34±0.43 D, −0.79±0.38 D, and−0.82±0.43 D, respectively. The Mid-EDOF group showed slower myopia progression by 0.48 D/year compared with the control group (59% suppression, *P*<0.01), whereas no significant effect was observed in the Low-EDOF group (Fig. 3).

**FIG. 4.**
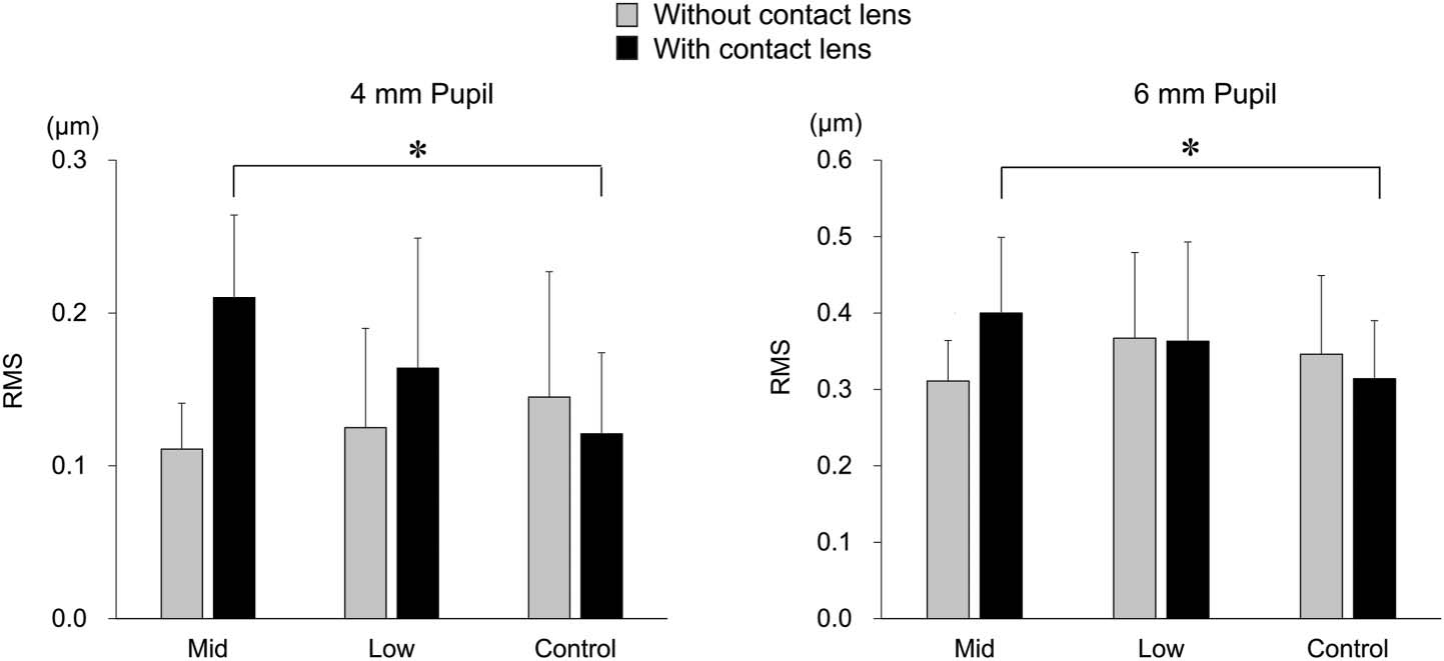
Total HOAs with 4-mm and 6-mm pupil. Higher-order aberrations were significantly increased in the Mid-EDOF group compared with the control group, whereas no significant difference was observed between the Low-EDOF and control groups. Mid: Mid-EDOF group, Low: Low-EDOF group; EDOF, Extended depth-of-focus; RMS, root mean square.

**FIG. 5.**
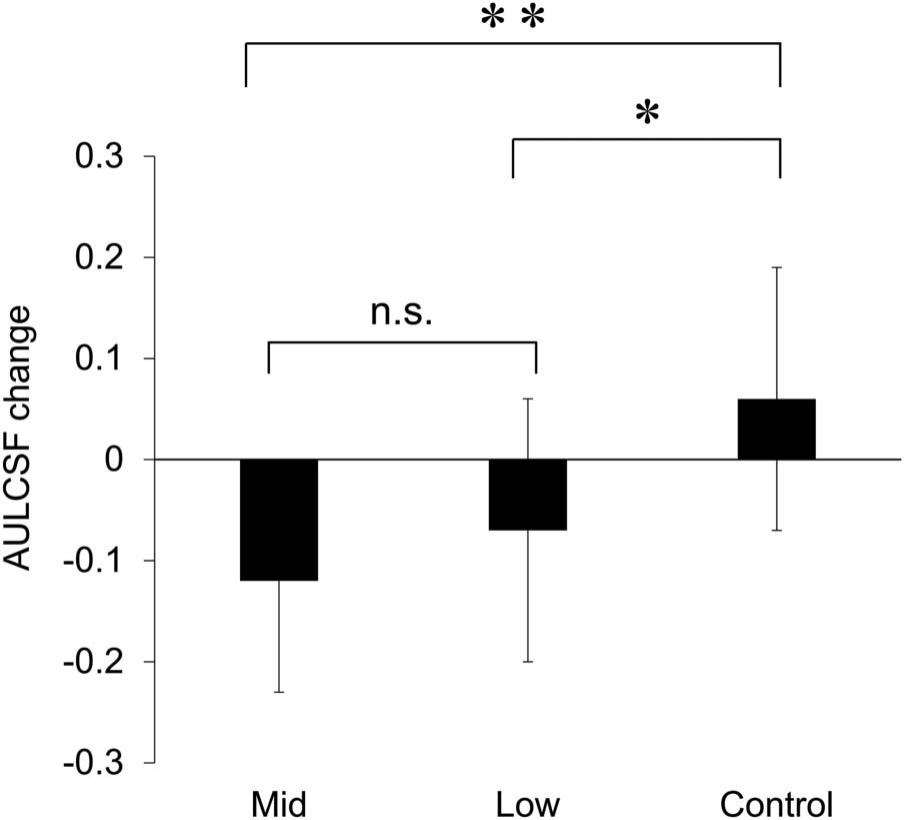
AULCSF change from spectacles to contact lenses. The AULCSF was significantly reduced in both EDOF groups compared with the control group, with no significant difference between the two EDOF groups. Mid: Mid-EDOF group, Low: Low-EDOF group; AULCSF, area under the log contrast sensitivity function; EDOF, Extended depth-of-focus; n.s., nonsignificant.

**FIG. 6.**
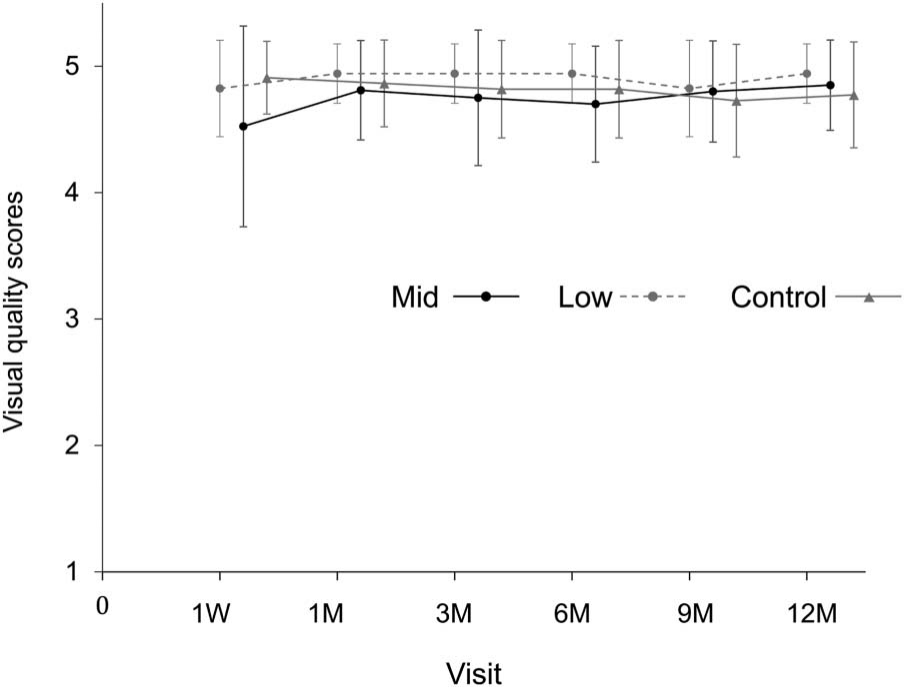
Subjective visual quality score. The subjective visual quality scores did not differ significantly among the groups during study period. Mid: Mid-EDOF group, Low: Low-EDOF group. EDOF, Extended depth-of-focus.

Under both 4- and 6-mm pupil conditions, HOAs without contact lens wear did not differ significantly among the groups (Table 2). With lens wear, HOAs increased significantly in the Mid-EDOF group compared with the control (*P*<0.01), with no difference between the Low-EDOF and control groups (Fig. 4). The AULCSF declined significantly with contact lens wear in the Mid-EDOF group compared with spectacle use (*P*<0.01). Compared with the control, both EDOF groups showed a significant reduction in the AULCSF (*P*<0.01 for both), with no difference between the Mid- and Low-EDOF groups (Fig. 5). Subjective visual quality scores did not differ significantly among the groups over time (Fig. 6).

**TABLE 2.** HOAs and AULCSF

		Mid	Low	Control
		(n=20)	(n=22)	(n=17)
HOAs 4-mm pupil (RMS μm)	Uncorrected	0.111±0.030	0.125±0.065	0.145±0.082
	With CL	0.210±0.054	0.164±0.085	0.121±0.053
HOAs 6-mm pupil (RMS μm)	Uncorrected	0.311±0.053	0.367±0.112	0.346±0.103
	With CL	0.400±0.099	0.363±0.130	0.314±0.076
AULCSF	With spectacles	1.49±0.08	1.44±0.12	1.38±0.17
	With CL	1.37±0.12	1.37±0.13	1.45±0.09
	Change from spectacles to CL	−0.12±0.11	−0.07±0.13	0.06±0.13

AULCSF, area under the log contrast sensitivity function; CL, contact lens; HOA, higher-order aberration; RMS, root mean square.

## DISCUSSION

This 12-month randomized controlled trial investigated the effect of the differences in optical design of EDOF soft contact lenses on myopia control and visual function in Japanese children. Lenses with a stronger EDOF profile (Mid-EDOF) significantly suppressed axial elongation and SE progression compared with the single-vision control lenses, whereas Low-EDOF lenses did not show statistically significant effects. These findings indicate that the degree of EDOF is a key determinant of the efficacy of EDOF lens designs. The difference in treatment efficacy observed between the Mid- and Low-EDOF lenses may be attributed to differences in the magnitude of the EDOF profile, as illustrated by their respective power distribution patterns. A greater variation in optical power may enhance myopia control efficacy but also increase optical side effects, such as reduced contrast sensitivity and increased HOAs. The Mid-EDOF group also exhibited significant SE suppression at 12 months but not at 6 months, suggesting that at least 1 year of lens wear may be necessary for meaningful changes in the SE. This observation is consistent with the findings of previous studies on progressive addition lens, in which higher addition powers achieved stronger myopia control.^23^

The strengths of this study include its randomized controlled design, use of daily disposable lenses suitable for real-world pediatric use, and comprehensive assessment of refractive and visual function. To the best of our knowledge, this is the first randomized controlled study to evaluate EDOF lens design variation in Japanese children, providing timely clinical evidence. As of January 2026, the dual-focus myopia control lens (MiSight 1 day, CooperVision) is the only approved soft contact lens for myopia control in Japan. The Mid-EDOF lens achieved efficacy comparable to that of the dual-focus myopia control lens, while showing similar trends in the effect on visual quality.^37–39^ Several features of the present study distinguish it from previous studies of myopia-control soft contact lenses. First, we directly compared two EDOF lens designs that were identical in material and general lens characteristics but differed in the magnitude of depth-of-focus extension, allowing a more focused evaluation of the role of the optical profile itself. Second, both myopia control efficacy and multiple aspects of visual function were assessed simultaneously. These findings provide clinically relevant insights into how variation in EDOF design influences the balance between treatment efficacy and visual performance. In addition, evaluation of treatment adherence using a randomized controlled design further enhances the clinical relevance of our findings.

The myopia control effect observed in the Mid-EDOF group (49% suppression in axial elongation and 59% in SE progression) is consistent with the findings of previous studies on EDOF lenses.^26^ For example, in Indian children, Manoharan et al.^26^ reported a 49% reduction in axial elongation and 59% reduction in SE progression over 1 year with Mid-EDOF lenses, compared with single-vision spectacles. In the current study, notably, the stronger EDOF profile of the Mid-EDOF group was associated with measurable changes in visual function, including reduced contrast sensitivity and increased HOAs under mesopic conditions. These findings highlight the clinical trade-off between myopia-control efficacy and visual quality, which is broadly consistent with that reported for other EDOF and multifocal myopia control designs, supporting the generalizability of this relationship across optical strategies. However, these objective degradations were not accompanied by a decline in the subjective visual quality scores. This discrepancy may reflect the relatively high tolerance of children to mild visual degradation in daily life, particularly when the correction is perceived as acceptable or improved compared with their habitual vision. Although high-addition multifocal lenses are known to degrade visual quality despite enhanced efficacy,^23,39^ our findings are consistent with those previous studies, including dual-focus myopia control lenses (MiSight 1 day),^40^ suggesting that children may accept certain optical compromises in exchange for a greater treatment benefit. Although HOA metrics provide useful information on optical quality, they may not fully reflect real-world visual performance or patient-perceived visual function. Therefore, interpretation of these metrics in isolation should be approached with caution. In this context, contrast sensitivity and subjective visual quality are considered more clinically relevant indicators, as they better represent functional vision in daily life.

From a practical perspective, lens fitting and treatment adherence are important determinants of real-world myopia-control outcomes. In the present study, lens fitting centration was comparable among groups, and the annular optical design of the EDOF lenses may be relatively tolerant to minor decentration. Reduced wearing time in some participants may have been related to early lens removal after returning home or before bathing, particularly in younger children. In addition, some participants reported symptoms of dryness in the evening, which may be associated with the hydrogel lens material and could have contributed to shorter wearing times. These findings highlight the importance of adherence in achieving optimal myopia control outcomes in real-world settings. Further evaluation of the relationship between wearing time and myopia-control efficacy could provide valuable insights into the treatment “dose” effect in real-world use. However, this analysis was not included in the present study because of the limited sample size and insufficient statistical power. Future studies with larger cohorts are warranted to better clarify the dose–response relationship.

Although the observation period of the present study was relatively short (1 year), no serious adverse events occurred, and the corneal endothelial cell density remained stable across all groups over 12 months. These findings are consistent with Lam et al.'s study investigating long-term safety, which found that 10-year daily wear of hydrogel MiSight contact lenses had no significant effect on corneal endothelial cell density in children.^41^ Although the oxygen permeability of the EDOF contact lenses is slightly higher than that of dual-focus myopia control lenses (MiSight 1 day), further long-term investigations into their corneal safety, similar to those conducted for the latter, are warranted.

The limitations of this study include the relatively small sample size for subgroup analyses and the short follow-up period of 12 months, which precluded evaluation of long-term durability. The simplicity of the five-point subjective visual quality scale may not capture subtle differences. Previous studies have suggested that rebound effects vary by treatment modality. Dual-focus myopia control lenses have not been shown to induce accelerated myopia progression even after up to 6 years of use,^23^ whereas low-dose atropine shows greater post-treatment acceleration at higher concentrations.^13^ However, because the follow-up period in the present study was limited to 1 year, rebound effects could not be assessed. Individual baseline characteristics such as refractive error and AL may influence treatment response. These factors should be further investigated in larger, adequately powered studies. In addition, as astigmatism restricted to 0.75 D or less was set as an inclusion criterion, the efficacy and visual function outcomes of EDOF contact lenses in patients with higher degrees of astigmatism could not be determined.

In conclusion, EDOF soft contact lenses represent a promising option for myopia control in Japanese children, with treatment efficacy depending on the optical profile. Mid-EDOF lenses provided greater suppression of myopia progression compared than that with Low-EDOF and single-vision lenses, with acceptable vision quality. Further research should be aimed at refining lens designs to better balance efficacy with visual performance, explore the long-term effects, and support personalized myopia-control strategies.
